# Behind the Non-Uniform Breakup of Bubble Slug in Y-Shaped Microchannel: Dynamics and Mechanisms

**DOI:** 10.3390/mi15060695

**Published:** 2024-05-24

**Authors:** Haoxiang Huang, Jiazheng Liu, Jialing Yu, Wentao Pan, Zhe Yan, Zhenhai Pan

**Affiliations:** 1 School of Mechanical Engineering, Shanghai Jiao Tong University, Shanghai 200240, China; p461265034@sjtu.edu.cn (H.H.); jiazheng_liu@alumni.sjtu.edu.cn (J.L.); yujialing@sjtu.edu.cn (J.Y.); 2 Earth, Ocean and Atmospheric Sciences, The Hong Kong University of Science and Technology (Guangzhou), Guangzhou 511453, China; 3 Shanghai Institute of Technical Physics, Chinese Academy of Sciences, Shanghai 200083, China; 4 School of Mechanical Engineering, Shanghai Institute of Technology, Shanghai 201418, China; panzhh_sit@163.com

**Keywords:** non-uniform breakup, bubble slug, breakup patterns, volume distribution, numerical study

## Abstract

Bubble flow in confined geometries is a problem of fundamental and technological significance. Among all the forms, bubble breakup in bifurcated microchannels is one of the most commonly encountered scenarios, where an in-depth understanding is necessary for better leveraging the process. This study numerically investigates the non-uniform breakup of a bubble slug in Y-shaped microchannels under different flow ratios, Reynolds numbers, and initial bubble volumes. Overall, the bubble can either breakup or non-breakup when passing through the bifurcation and shows different forms depending on flow regimes. The flow ratio-Reynolds number phase diagrams indicate a power–law transition line of breakup and non-breakup. The bubble takes longer to break up with rising flow ratios yet breaks earlier with higher Reynolds numbers and volumes. Non-breakup takes less time than the breakup patterns. Flow ratio is the origin of non-uniform breakup. Both the Reynolds number and initial volume influence the bubble states when reaching the bifurcation and thus affect subsequent processes. Bubble neck dynamics are analyzed to describe the breakup further. The volume distribution after breaking up is found to have a quadratic relation with the flow ratio. Our study is hoped to provide insights for practical applications related to non-uniform bubble breakups.

## 1. Introduction

Bubble flow in confined geometries is a common but important phenomenon. It plays versatile roles in both our daily lives and industries, including medicine [1], petrochemicals [2], cosmetics [3], food [4], and cooling technologies [5,6]. Under different situations, it is desirable to either leverage or suppress the bubble behaviors, from which an in-depth understanding of the process becomes necessary.

One major application for bubble flow is obtaining highly repeatable and monodispersed microbubbles with controllable sizes. This process is commonly realized by passively breaking bubbles through bifurcations or obstacles [7]. Previous studies revealed that the influencing factors and mechanisms of bubble breakup behaviors, e.g., patterns, evolution, transition, and volume distribution, can be complicated [8,9,10]. Moreover, due to the small dimensions, factors that are insignificant at macro scales may become important [11].

Physically, the breaking up of bubbles in microchannels is similar to droplets [12,13,14,15], suggesting that many results on droplet breakup may also apply to bubbles [16,17,18]. In particular, the dominant factors of bubble breakup include the gas/liquid flow rates, channel/bubble dimensions, channel shape/materials, and fluid properties. Fu et al. [19] investigated the breakup dynamics of N_2_ bubbles in glycerol–water mixtures at a symmetric T-junction. Four flow regimes were observed, i.e., the bubble broke up in the obstructed liquid, a gap appeared between the bubble and walls after a certain time, a gap permanently existed between the bubble and walls, and non-breakup. The patterns were revealed to be controlled by gas and liquid flow rates and viscosity, and the transition of breakup and non-breakup followed a power–law relationship between bubble extension and capillary number. Liu et al. [20] studied the effects of the width ratio between the main and branch channels on bubble breakup in a T-junction. It was found that the bubble experienced non-breakup, tunnel breakup, and obstructed breakup with the rising width ratio, and the breakup process could be divided into extension, squeeze, and pinch-off stages. They also observed that the non-breakup process underwent two stages: extension and pushing. Nagargoje and Gupta [9] conducted experiments using three Newtonian liquids and explored the N_2_ bubble dynamics under different capillary (*Ca*, 0.00078–0.044) and Reynolds (*Re*, 3.5–127) numbers when facing a Y-shaped Polydimethylsiloxane (PDMS) bifurcating channel. Both breakup and non-breakup were reported, where the Reynolds number and bubble length played a major role in the transition of the two patterns. An asymmetrical behavior during bubble breakup was observed, which was explained as being derived from channel imperfections and flow instability. To gain more details on the gas–liquid interface during bubble breakup, Lu et al. [21] quantitatively studied the interfacial dynamics of bubble breakup at a T-junction. It was identified that a critical bubble neck width existed, below which (i.e., minimum bubble neck width < critical bubble neck width) the breakup was irreversible and otherwise reversible. The minimum bubble neck width was disclosed to have a power–law relation with the remaining time. Similar results were reported in the study afterward [22,23]. However, obtaining flow details through experiments on a micro-scale can be tricky and is accompanied by large relative errors, affecting deeper insights behind the phenomena.

Numerical studies allow for comprehensively displaying the distribution of key physical quantities during bubble breakup, investigating factors that are difficult or expensive in conducting experiments, and therefore promoting perceptions of the dynamic mechanism of the process [24,25,26,27,28]. Lou et al. [29] simulated the bubble behaviors when passing through a Y-junction under different channel wettability, liquid viscosity, initial bubble volume, and flow ratio of the two branches by the lattice Boltzmann method (LBM). The results showed that higher hydrophilicity, lower liquid viscosity, and larger bubble size are conducive to bubble breakup, and the demarcation between breakup and non-breakup follows an exponential relation between the flow ratio and capillary number. Zhang et al. [30] studied the bubble breakups in a multi-level tree-shaped microchannel through the two-dimensional volume of fluid (VOF) method. They characterized the pressure evolution at the center of bifurcation under different levels and inlet capillary numbers, which served as evidence, showing that bubbles in the lower-level bifurcations tended to be unsteady and would lead to chaos in the higher-level bifurcations. Zhang et al. [31] studied bubble breakup dynamics in a microchannel consisting of two T-junctions using the VOF method, where different flow regimes under *Ca* = 0.001–0.008 and bubble lengths were demonstrated.

Despite the ubiquitousness and wide range of applications in practice, especially in the medical field, few studies have focused on non-uniform bubble breakup in bifurcating microchannels. Chen et al. [32] numerically studied the effects of gravity, inertia, and surface tension on bubble flow and pressure and shear stress gradients for concern of potential damage to the pulmonary epithelium when mechanical ventilation was used for lung therapy. The results indicated that the asymmetric bubble propagation would lead to higher pressure and shear stress gradients along the Y-junction than symmetric cases, and both inertia and surface tension play a major role in determining the pressure gradients. Gas embolotherapy is a potential cancer treatment that employs intra-arterial bubbles to occlude tumor blood supply. However, when the bubbles pass through the bifurcation of blood vessels, they may inhomogeneously break up, thus resulting in unfavorable bioeffects. Qamar et al. [33] modeled the breakup behavior through the VOF method to learn more process details. The model identified three flow regimes (homogeneous, nonhomogeneous, and reversal breakup) and indicated that the shear forces induced by vortices near the bifurcation should be responsible for endothelial cell damage on the vessel walls. The non-uniform bubble breakup remains an open question.

In this study, we numerically investigate the non-uniform breakup of a bubble slug in Y-shaped microchannels. The VOF method is employed to capture the bubble–liquid interface and is validated against the experimental results. We first introduce several observed patterns of non-uniform breakup, then discuss the different roles that the flow ratio, Reynolds number, and initial bubble volume play. The breakup process is further described by bubble neck dynamics, where squeezing, transition, and pinch-off stages are identified. Finally, the volume distribution of the non-uniform breakup is discussed. Our study is expected to provide insights into bubble breakup and applications based on this phenomenon.

## 2. Methodology

### 2.1. Problem Statement

**Figure 1:**
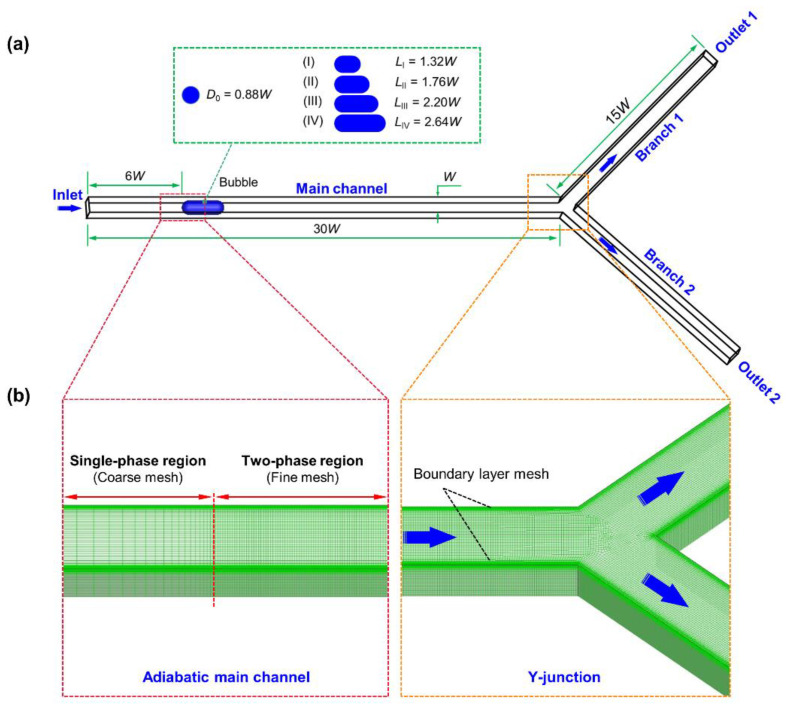
(**a**) Illustration of the physical model of the present study; (**b**) mesh details at the main channel and Y-shaped junction.

The non-uniform breakup of the isolated bubble is induced by unbalanced outlet flow rates when passing through a Y-shaped square microchannel (Figure 1). The channel width and height are set as W = 400 μm, and the length of its main part is 30 W. To ensure a complete breakup process, we set the length of the two branches to 15 W. The bifurcation angle of the two branches is *π*/2 in radians.

A single-phase steady-state water flow is designated for initialization. Then, a seed bubble (air) with a cross-section diameter *D*_0_ = 0.88 W and a length *L* = 1.32 W–2.64 W (i.e., 1.5*D*_0_–3.0*D*_0_) is patched on the channel center, 6 W from the inlet (Figure 1a). The channel inlet is set as a pressure inlet with a value of 100 kPa, and the two outlets are set as velocity outlets. The working temperature is 293 K. The physical properties of water and air are shown in Table 1.

### 2.2. Model and Implementation

In this work, we employ the finite volume method to perform numerical simulations. The VOF method is used to track the water–bubble interface. By setting air as the tracked phase and *α* as its volume fraction in the mesh cell, cells with *α* = 1 are identified as the bubble, *α* = 0 as water, and 0 < *α* < 1 as the interface between the two phases. Tracking of the interface between the phases is accomplished by solving the continuity equation:

(1) $$\frac{\partial \alpha }{\partial t}+U\cdot \nabla \alpha =0$$

where *t* denotes time, and ***U*** denotes the flow velocity. With Equation (1), the liquid and gas phases are mass-conserved, and the two-phase flow problem can be solved through single-phase formulations [35,36].

The flow inside the microchannel is considered Newtonian and laminar; therefore, we have the continuity and momentum equations as follows:

(2) $$\frac{\partial \rho }{\partial t}+\nabla \left(\rho U\right)=0$$

(3) $$\frac{\partial \left(\rho U\right)}{\partial t}+\nabla \cdot \left(\rho U\cdot U\right)=-\nabla p+\nabla \left[\mu \left(\nabla U+\nabla U^{T}\right)\right]+F_{s}$$

where *ρ* is the density, *p* the pressure, *μ* viscosity, and ***F****_s_* the surface tension. The fluid properties are determined by the volume fraction of each phase, namely:

(4) $$\rho =\alpha \cdot \rho_{air}+(1-\alpha )\cdot \rho_{water}$$

(5) $$\mu =\alpha \cdot \mu_{air}+(1-\alpha )\cdot \mu_{water}$$

Based on the continuum surface force (CSF) model [37,38], the surface tension ***F****_s_* is treated as a volumetric body force that acts in the cells around the interface with a volume fraction gradient ∇*C* not equal to zero:

(6) $$F_{s}=\sigma \kappa \delta_{s}n=-\sigma \nabla \cdot \left(\frac{\nabla C}{\left|\nabla C\right|}\right)\left|\nabla C\right|$$

Here, *σ* is the fluid surface tension coefficient, *κ* is the interface curvature (∇·(∇*C* /|∇*C*|), *δ_s_* is a coefficient containing the Kronecker delta function, and ***n*** is the unit normal vector at the interface (∇*C*/|∇*C*|). An alternative surface tension modeling approach involves treating ***F****_s_* directly as an interfacial force [39], which may yield higher fidelity under certain conditions. Considering computational efficiency, we have opted for the CSF method. Furthermore, mesh refinement is employed at the acute angles of the Y-junction to ensure that the numerical calculations of the volume fraction gradient and interface normal remain stable and accurate at these critical locations.

We process the time and bubble volume into dimensionless forms as follows:

(7) $$t^{*}=\frac{tU_{ave}}{W},\;V^{*}=\frac{6V}{\pi W^{3}}$$

where *U_ave_* represents the average inlet velocity, and *V* denotes the bubble volume. The initial bubble volume is denoted by *V*_0_^*^. The Reynolds number (*Re*) is defined as

(8) $$Re=\frac{\rho_{water}U_{ave}W}{\mu_{water}}$$

In this study, the non-uniform breakup of the bubble is realized by setting different outlet flow rates for the two branches, which is characterized by the flow ratio *γ*:

(9) $$\gamma =\frac{U_{ave,1}}{U_{ave,2}}$$

where *U_ave,_*_1_ and *U_ave,_*_2_ represent the average outlet velocity at the upper and lower branch channels (Figure 1).

The equations we used to calculate the total bubble volume and average outlet velocity are as follows:

(10) $$V_{b}=\int CdV$$

(11) $$\bar{U}=\frac{1}{A}\int UdA$$

The model geometry and generated mesh are shown in Figure 1a and Figure 1b, respectively. In the present study, we adopt structured meshes and gradually refine them when approaching the wall edge. The mesh near the boundary layer is ensured to have more than five cells so that the liquid film near this region can be accurately captured [40,41]. To improve computational efficiency, we divide the microchannel into two symmetrical parts and calculate one of them. The calculation is performed by the 28-core workstation (processor: Intel Xeon E5-4660 v3). A no-slip boundary condition is used on the channel wall, and the static contact angle between water and the channel wall is assumed as 0°, suggesting the liquid phase can fully wet the wall. The PISO algorithm is employed for pressure-velocity coupling. PRESTO and QUICK schemes are used to discretize pressure and momentum, respectively. The Green–Gauss node-based method calculates scalar gradients, and the Geo-Reconstruct method for volume-fraction discretization is used to maintain a high resolution of the interface. The residual values for continuity and the *x*-, *y*-, and *z*-velocities are set as 1 × 10^−5^. During the calculation, small errors in the volume fraction gradient may lead to spurious currents derived from surface tension not strictly perpendicular to the interface at a low Reynolds number. Here, we employ a moving reference frame (1 m/s) to suppress the influence of spurious currents on the simulation results [35]. The global Courant number is ensured to <0.1 by setting a constant time step value ranging from 1 × 10^−8^ s to 3 × 10^−7^ s.

### 2.3. Validation

This section first validates model effectiveness and then mesh independence.

**Figure 2:**
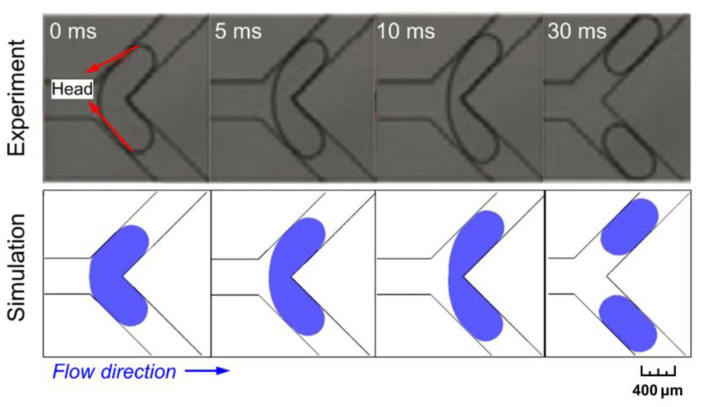
Validation of the present model: comparison between our simulation and experimental results from the literature [42].

A comparison between our numerical outputs and experimental results [42] is made by concerning a similar situation to the present study, i.e., bubble breakup in a Y-shaped microchannel. The cross-section of the channel is rectangular, with 400 × 50 μm in width and height, and the length of the two branches is 10 mm (bifurcation angle: *π*/2). In the experiment, bubbles are generated at the channel inlet by pumping the liquid (an aqueous solution with 0.3% sodium dodecyl sulfate) and gas (nitrogen) at flow rates of 2.2 mL/h and 2.4 mL/h, respectively. The validation simulation conditions are the same as the experimental setup. Three-dimensional modeling is conducted to simulate the bubble breakup process at the Y-junction, and the results agree well with the experiments (Figure 2).

A mesh independence verification is implemented with cell numbers ranging from 499,890 to 2,401,453. The inlet Reynolds numbers are 100 and 600, and the initial bubble length is 2.64 W. We compare the bubble length (in dimensionless form, *L*/*W*) when its nose has just arrived at the Y-junction and set the value when the cell number = 1,003,170 as the reference value (r.v.). The deviation under different mesh resolutions is calculated as |deviation| = |(bubble length-r.v.)/(r.v.)|. As shown in Table 2, when the cell number reaches 1,003,170, further refinement of the mesh hardly changes the result, with a deviation smaller than 0.41%. Therefore, we adopt 1,003,170 cells in the simulation.

## 3. Results and Discussion

### 3.1. Non-Uniform Breakup Patterns of the Bubble

**Figure 3:**
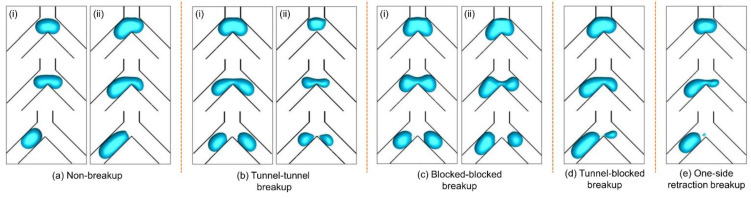
Non-uniform breakup patterns of a bubble traveling through a Y-shaped microchannel: (**a**) non-breakup; (**b**) tunnel–tunnel breakup; (**c**) obstruction–obstruction breakup; (**d**) obstruction–tunnel breakup; (**e**) one-side retraction breakup.

We first introduce several definitions that characterize bubble dynamics. Generally, a bubble can breakup or non-breakup when going through a bifurcation; afterward, its flow regime shows a tunnel flow or blocked flow [43]. Here, tunnel and blocked are two purely geometrical descriptions, which indicate whether a visible gap between the bubble and channel wall exists (tunnel flow) or not (blocked flow). In this study, five flow regimes are identified under different flow ratios (*γ* = 1.0–4.0), Reynolds numbers (*Re* = 100–600), and initial bubble volumes (*V*_0_^*^ = 1.2–2.7) (Figure 3):

(a) Non-breakup. As the bubble arrives at the Y-junction, it first slows down and slightly penetrates the junction, then flows into one of the branches under a random disturbance (Figure 3a(i)). If the flow ratio is large enough, the bubble may also directly enter the high-flow-rate branch without breakup (Figure 3a(ii)).
(b) Tunnel–tunnel breakup. Under a synergy effect of squeezing force, shearing force, and the sharp corner of the Y-junction, the bubble can continue to deform and finally break up [2]. A tunnel–tunnel breakup pattern depicts that both bubble-laden flows in the two branches show a tunnel style after the initial bubble breaks up at the bifurcation. The breakup can be either symmetrical (Figure 3b(i)) or asymmetrical (Figure 3b(ii)).
(c) Blocked–blocked breakup. After breaking up at the bifurcation, the daughter bubbles may keep blocking the two branch channels. This situation often happens with large-volume bubbles. The breakup can be either symmetrical (Figure 3c(i)) or asymmetrical (Figure 3c(ii)).
(d) Tunnel–blocked breakup. Tunnel flow in one branch channel and blocked flow in the other (Figure 3d).
(e) One-side retraction breakup. When the flow ratio is relatively large, a daughter bubble about to separate from the initial bubble may be retracted back into the initial bubble due to the surface tension, during which a satellite bubble is generated. The main and satellite bubbles will enter the high-flow-rate branch (Figure 3e).

**Figure 4:**
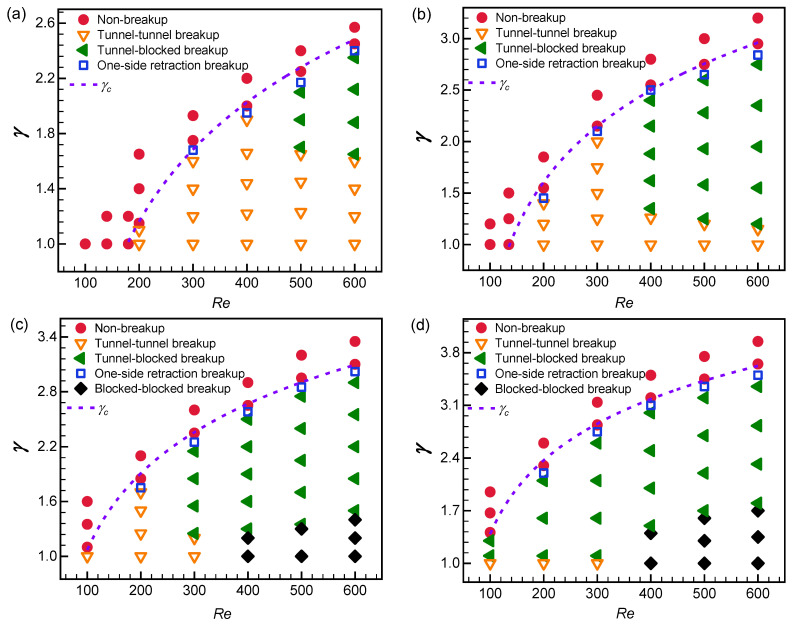
Phase diagram of bubble breakup patterns under different initial bubble volumes (*V*_0_^*^): (**a**) *V*_0_^*^ = 1.2; (**b**) *V*_0_^*^ = 1.7; (**c**) *V*_0_^*^ = 2.2; (**d**) *V*_0_^*^ = 2.7. Solid circle: non-breakup pattern; open down triangle: tunnel–tunnel breakup pattern; solid left triangle: tunnel-blocked breakup pattern; open square: one-side retraction breakup pattern; solid diamond: blocked–blocked breakup pattern.

The phase diagrams for the above patterns are plotted in Figure 4. Overall, the non-breakup pattern tends to appear under a small Reynolds number, and as the Reynolds number grows, it appears at high flow ratios. The transition line of non-breakup and breakup patterns conforms to a power–law relationship (flow ratio versus Reynolds number, dash line in Figure 4):

(12) $$\gamma_{c}=\ln(c_{1}\cdot Re+c_{2})$$

where *γ_c_* is the critical flow ratio, and *c*_1_ and *c*_2_ are two fitting parameters whose values are listed in Table 3. Parameter *c*_1_ shows an increasing trend as the bubble volume extends, suggesting that larger bubbles in the Y-shaped microchannels require a higher flow ratio to trigger the non-breakup pattern under the same Reynolds number. It is worth noting that these parameters are feasible under fixed channel geometry, and their value may be varied with different channel geometry.

The scene of the one-side retraction pattern is close to that of the non-breakup pattern. This pattern occurs under a slightly lower flow ratio than the non-breakup pattern and a Reynolds number of at least 200 (Figure 4), which indicates that the one-side retraction pattern is susceptible to the flow ratio and Reynolds number and thus can be considered as a transitional state between bubble breakup and non-breakup.

The tunnel–tunnel breakup pattern commonly occurs at small flow ratios and Reynolds numbers and is the dominant pattern for small bubbles, e.g., those with *V*_0_^*^ = 1.2 (Figure 4a). As the initial bubble volume increases to 1.7, this pattern is still dominant at small Reynolds numbers, typically when *Re* ≤ 300; however, when *Re* ≥ 400, it only appears under *γ* ≈ 1.0–1.26 (Figure 4b). Similarly, when the bubble volume extends to 2.2, the tunnel–tunnel pattern is most likely to occur when *Re* ≤ 200, while when *Re* = 300, it only occurs at *γ* = 1.0–1.2 (Figure 4c). As the initial bubble volume reaches 2.7, the tunnel–tunnel breakup pattern occurs at *Re* = 100–300, *γ* = 1.0 (Figure 4d).

The tunnel-blocked pattern is a typical manifestation of non-uniform breakup. This pattern shows up under large Reynolds numbers and high flow ratios. For instance, when the initial bubble volume is 1.2, it takes place at *Re* = 500, *γ* = 1.7–2.1, and *Re* = 600, *γ* = 1.65–2.35 (Figure 4a). As the bubble volume further grows, the tunnel-blocked breakup pattern gradually becomes the dominant regime, especially when *V*_0_^*^ ≥ 2.2 (Figure 4c,d). The flow ratio should be larger than 1.0 to prompt this pattern.

The blocked–blocked pattern appears when a large bubble breaks up. In the concerned range of bubble volumes, this pattern only occurs when *V*_0_^*^ ≥ 2.2, and it prefers a high Reynolds number (*Re* ≥ 400) and relatively low flow ratio (*γ* ≈ 1.0–1.7) (Figure 4c,d).

### 3.2. Effects of Flow Ratio, Reynolds Number, and Bubble Volume on Breakup Dynamics

**Figure 5:**
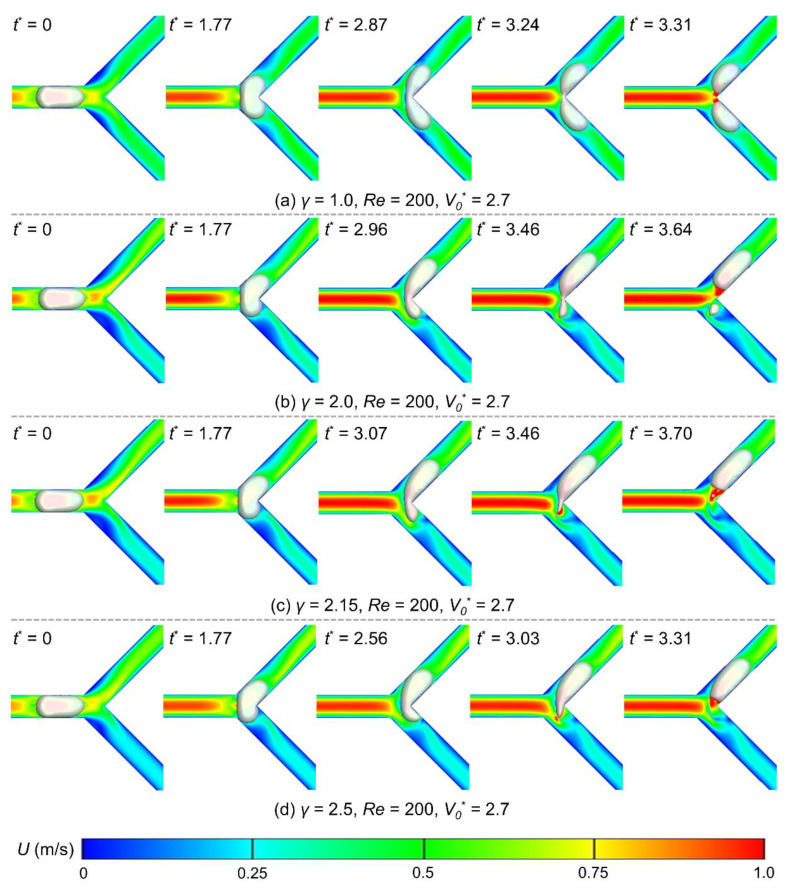
Snapshots of bubble behaviors when passing the Y-junction under different flow ratios: (**a**) *γ* = 1.0; (**b**) *γ* = 2.0; (**c**) *γ* = 2.15; (**d**) *γ* = 2.2. The Reynolds number is 200, and the initial bubble volume is 2.7. Each contour map represents the velocity field at the corresponding moment.

This section further discusses the flow ratio, Reynolds number, and bubble volume effects on the breakup dynamics at the Y-junction. The unbalanced flow rate in the two branches should be the critical factor for non-uniform breakups. Figure 5 displays the bubble evolution at different flow ratios (*γ* = 1.0–2.5), where the Reynolds number is set as 200, and the initial bubble volume is 2.7. Under these conditions, the flow regime should be in four distinct patterns (suggested in Figure 4d). We define t^*^ = 0 as the instant the bubble nose arrives at the Y-junction and note that the same Reynolds number indicates the same flow velocity in the main channel (Equation (8)). Therefore, the time for the bubble to fully enter the bifurcation is also the same, all at t^*^ = 1.77.

The bubble breaks up in a symmetric tunnel-flow manner at t^*^ = 3.31 when the flow rates in the two branches are equal (*γ* = 1.0, Figure 5a). As the flow ratio increases, it asymmetrically enters the two branches due to the unbalanced flow rate, with more volume moving into the high-flow-rate branch. The bubble breaks up in a tunnel-blocked pattern at t^*^ = 3.64 when the flow ratio is 2.0, slightly delayed compared to the *γ* = 1.0 case (Figure 5b). When the flow ratio rose to 2.15, the bubble showed a one-side retraction pattern after fully entering the bifurcation. Eventually, it generates a satellite bubble following the main bubble into the high-flow-rate branch at t^*^ = 3.70 (Figure 5c). As the flow ratio reaches 2.5, the bubble presents a non-breakup pattern and entirely enters the branch at t^*^ = 3.31 (Figure 5d). The above results on the breakup times reveal that a bubble takes longer to break up with a growing flow ratio; in contrast, the period is shortened if the flow ratio is high enough to trigger the non-breakup pattern. This phenomenon is due to the significantly reduced flow rate (compared with the *γ* = 1.0 case) in the lower branch under large flow ratios, where the fluid cannot apply sufficient shear forces to break the bubble. The non-breakup process takes less time than the breakup process, as it does not rupture the bubble–liquid interface.

**Figure 6:**
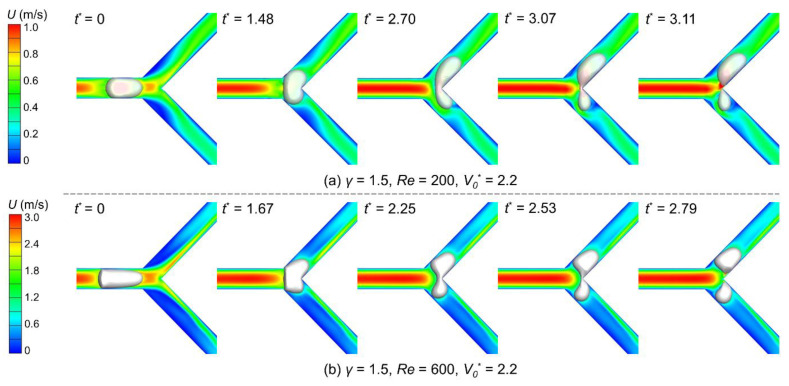
Snapshots of bubble behaviors when passing the Y-junction under different Reynolds numbers: (**a**) *Re* = 200 (Detailed bubble breakup process can be seen in the Supplementary Material Video S1); (**b**) *Re* = 600. The flow ratio is 1.5, and the initial bubble volume is 2.2. Each contour map represents the velocity field at the corresponding moment.

We then investigate the effect of the Reynolds number on bubble breakup. Figure 6 shows bubble behaviors when the Reynolds number equals 200 and 600 while the flow ratio is 1.5 and the bubble volume is 2.2. When arriving at the Y-junction, the bubble flowing under a high Reynolds number is longer than under a low Reynolds number caused by the higher fluid drag (Figure 6, t^*^ = 0). Owing to this length difference, the longer bubble takes more time to enter the bifurcation completely, e.g., at t^*^ = 1.67 when *Re* = 600 verses t^*^ = 1.48 when *Re* = 200. However, it should also be mentioned that the longer bubble has more parts penetrated into the bifurcation at the moment. When *Re* = 600, the bubble tail profile has already transformed from a convex shape to a concave shape at t^*^ = 2.25, indicating an impending breakup [2], while at *Re* = 400, the transformation does not appear at least until t^*^ = 3.07. This discrepancy suggests an earlier bubble breakup under a higher Reynolds number and can be proved by the last snapshot in Figure 6a,b (t^*^ = 2.79 versus 3.11 for *Re* = 600 and 200, respectively).

**Figure 7:**
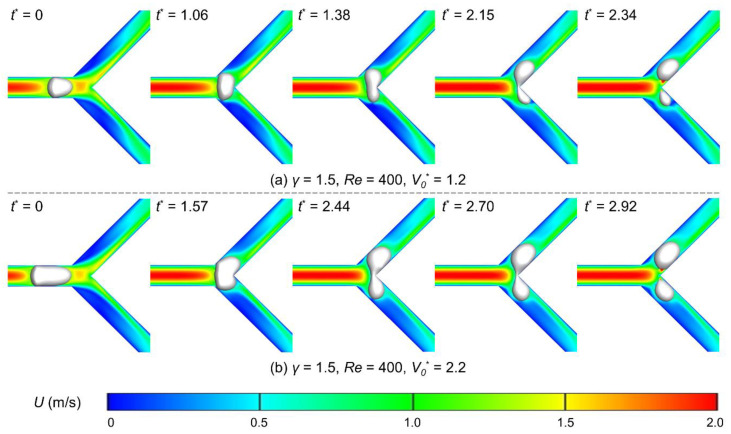
Snapshots of bubble behaviors when passing the Y-junction under different initial volumes: (**a**) *V*_0_^*^ = 1.2; (**b**) *V*_0_^*^ = 2.2. The flow ratio is 1.5, and the Reynolds number is 400. Each contour map represents the velocity field at the corresponding moment.

Finally, the influence of the initial bubble volume on breakup dynamics is studied. Cases of the Reynolds number 400, the flow ratio of 1.5, and the initial volumes of 1.2 and 2.2 are adopted for exemplification (Figure 7). As expected, a larger bubble requires more time to fully enter the junction, where the dimensionless time interval is 1.57 for the *V*_0_^*^ = 2.2 and 1.06 for the *V*_0_^*^ = 1.2. The nose of the larger bubble invades the sharp corner after fully entering the junction, yet the smaller bubble does not, implying that the larger bubble has initially entered the breakup process. As the bubble further deforms, the smaller bubble breaks up in a shorter period (at t^*^ = 2.34, compared with t^*^ = 2.92 of the larger bubble). Here, we note that this time difference does not imply small bubbles break up faster. Further discussion will be provided in Section 3.3.

### 3.3. Bubble Neck Dynamics

**Figure 8:**
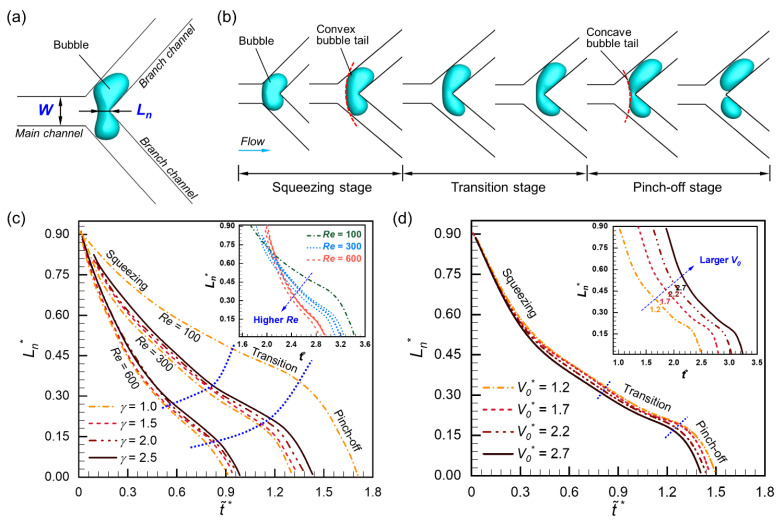
Illustration of bubble breakup process: (**a**) characterization parameters; (**b**) breakup stages; (**c**) evolution of bubble neck thickness under different *γ* and *Re* while *V*_0_^*^ = 2.7; (**d**) evolution of bubble neck thickness under different *V*_0_^*^ while *γ* = 1.5, *Re* = 400.

Bubble neck dynamics can further describe the breakup process by analyzing the bubble neck thickness *L_n_* (Figure 8a). Figure 8b shows a typical breakup process of the bubble, which can be divided into squeezing, transition, and pinch-off stages, depending on the evolution of neck thickness and the shape of the bubble tail [2]. The bubble tail is convex in the squeezing stage, gradually transforms to concave during the transition stage, and finally breaks up in the pinch-off stage. The specific variations in the bubble thickness under different flow conditions are depicted in Figure 8c and Figure 8d, respectively. Here, for convenience of comparison, we set $\tilde{t}$^*^ = 0 as the moment when the bubble fully enters the junction, where the thickness evolution with t^*^ is shown in the inserts. The bubble thickness is converted to a dimensionless form as:

(13) $${L_{n}}^{*}=\frac{L_{n}}{W}$$

Figure 8c plots the evolution of bubble thickness under different flow ratios and Reynolds numbers when the initial bubble volume is 2.7. Although the bubble breaks up in various patterns with an increasing flow ratio, the overall difference in the dynamics of the bubble thickness is limited, and the difference in the moment of breaking up is within 10% [= ($\tilde{t}$^*^|*_γ_* − $\tilde{t}$^*^|*_γ_*_=1_)/$\tilde{t}$^*^|*_γ_*_=1_]. In contrast, the Reynolds number plays a more significant role in bubble neck dynamics. As the Reynolds number increases, the bubble neck shrinks more rapidly, typically from $\tilde{t}$^*^ ≈ 1.7 when *Re* = 100 to $\tilde{t}$^*^ ≈ 1.35 when *Re* = 300, and to $\tilde{t}$^*^ ≈ 0.94 when *Re* = 600. The effect of the initial volume on bubble neck dynamics is presented in Figure 8d (*γ* = 1.5, *Re* = 400). As the bubble volume increases, the thinning rate of neck thickness rises slightly, suggesting that larger bubbles break up faster than smaller bubbles. The time difference at the breakup is within 5.8% [= ($\tilde{t}$^*^|*_V_*_0*=1.2_ − $\tilde{t}$^*^|*_V_*_0*_)/$\tilde{t}$^*^|*_V_*_0*=1.2_].

### 3.4. Volume Distribution after Breakup

**Figure 9:**
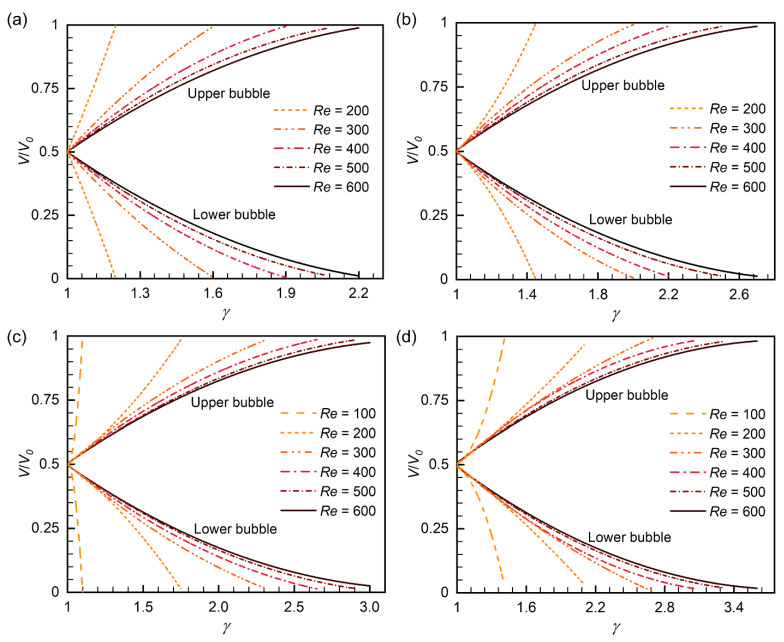
Volume distribution after bubble breakup at the Y-junction: (**a**) *V*_0_^*^ = 1.2, (**b**) *V*_0_^*^ = 1.7, (**c**) *V*_0_^*^ = 2.2, and (**d**) *V*_0_^*^ = 2.7. *V*_0_ denotes the initial bubble volume, and *V* denotes the volume of daughter bubbles.

The volume distribution after the bubble breaks up was investigated. Figure 9 shows the volumes of the two daughter bubbles under different flow ratios, Reynolds numbers, and initial bubble volumes, where the bubble volumes are converted to dimensionless forms as *V*/*V*_0_. As expected, a larger flow ratio leads to a more considerable difference in daughter bubble volumes, confirming its vital role in the non-uniform breakup. The volume of the upper bubble follows the following formula:

(14) $$V/V_{0}{|}_{\mathrm{upper}}=a\cdot \gamma^{2}+b\cdot \gamma +c$$

where *a*, *b*, and *c* are the fitting parameters given in Table 4. The volume of the lower bubble is thus *V*/*V*_0_|_lower_ = 1 − (*V*/*V*_0_|_upper_).

The Reynolds number also has an essential effect on bubble volume distribution. Under the same flow ratio and initial volume, the non-uniformity of bubble breakup decreases with the increasing Reynolds number. This phenomenon may be because bubbles under higher Reynolds numbers are more constrained by the symmetrical geometry (Figure 6). Moreover, it is also worth noting that as the Reynolds number increases, its influence on bubble volume distribution gradually decreases (Figure 9). The initial bubble volume has a similar effect on bubble volume distribution. The mechanism should also relate to the symmetrical geometry.

## 4. Conclusions

The present article numerically studied the non-uniform breakup of a bubble slug in Y-shaped microchannels. The simulations were performed by finite-volume schemes, and the VOF method was adopted for capturing the bubble–liquid interface. Innovatively, the non-uniform breakup behavior is driven by the active control of the flow rate in different branching channels. The model was validated against the experimental results and showed sufficient accuracy. Our calculations focused on the effects of the flow ratio (*γ*), Reynolds number (*Re*), and initial bubble volumes (*V*_0_^*^), covering the ranges of 1.0–4.0, 100–600, and 1.2–2.7, respectively. Under the concerned ranges, the bubble can either breakup or non-breakup, where the breakup differs in forms such as tunnel–tunnel breakup, blocked–blocked breakup, tunnel-blocked breakup, and one-side retraction breakup. The *γ*-*Re* phase diagrams were plotted, and the transition line between breakup and non-breakup conforms to a power–law relationship. The bubble slug took longer to break up with rising flow ratios, whereas it broke up earlier with higher Reynolds numbers and larger initial volumes. The non-breakup pattern took less time than the breakup patterns, as it did not rupture the bubble–liquid interface. Bubble neck dynamics were employed to further describe the breakup process by analyzing the bubble neck thickness, where squeezing, transition, and pinch-off stages were identified. After breaking up, the volume distribution was discussed and found to have a quadratic relation with the flow ratio. These results could provide a deeper understanding of the bubble breakup mechanisms and are hoped to help the design and optimization of microfluidic systems.

## Figures and Tables

**Table 1:** Physical properties of air and water at 293 K [34].

Physical Properties	Water	Air
Density, *ρ* (kg/m^3^)	998.2	1.205
Viscosity, *μ* (Pa·s)	1.005 × 10^−3^	1.81 × 10^−5^
Surface tension, *σ* (N/m)	0.07275	/

**Table 2:** Mesh independence verification.

Cell Number	499,890		1,003,170		1,586,530		2,401,453	
*Re*	100	600	100	600	100	600	100	600
Bubble length	2.122	2.563	2.196 (r.v.) ^†^	2.595 (r.v.) ^†^	2.187	2.589	2.190	2.592
|Deviation|	3.37%	1.23%	/	/	0.41%	0.23%	0.27%	0.12%

^†^ The abbreviation r.v. denotes reference value.

**Table 3:** Fitting parameters *c*_1_ and *c*_2_ in Equation (10) for different bubble volumes.

Fitting Parameter	*V*_0_^*^ = 1.2	*V*_0_^*^ = 1.7	*V*_0_^*^ = 2.2	*V*_0_^*^ = 2.7
*c*_1_	0.019	0.036	0.038	0.067
*c*_2_	−0.561	−2.177	−0.911	−2.691

**Table 4:** Fitting parameters a, b, and c in Equation (12).

V_0_^*^	Parameter	Re					
		100	200	300	400	500	600
1.2	*a*	\	1.38	−0.37	−0.30	−0.25	−0.21
	*b*	\	−0.54	1.78	1.42	1.22	1.08
	*c*	\	−0.34	−0.91	−0.62	−0.47	0.37
1.7	*a*	\	1.01	−0.20	−0.16	−0.14	−0.12
	*b*	\	−1.35	1.09	0.91	0.81	0.73
	*c*	\	0.85	−0.39	−0.25	−0.17	−0.10
2.2	*a*	50.01	0.30	−0.11	−0.10	−0.09	−0.09
	*b*	−99.94	−0.17	0.72	0.66	0.59	0.58
	*c*	50.43	0.37	−0.12	−0.06	−0.004	0.01
2.7	*a*	2.36	0.06	−0.06	−0.08	−0.06	−0.06
	*b*	−4.50	0.22	0.49	0.55	0.47	0.45
	*c*	2.65	0.22	0.06	0.03	0.10	0.12

## Data Availability

The data that support the findings of this study are available from the corresponding authors upon reasonable request.

## Supplementary Materials

The following supporting information can be downloaded at: https://www.mdpi.com/article/10.3390/mi15060695/s1, Video S1: Detailed bubble breakup process.mp4.
